# A Rare Association: Hereditary Hemorrhagic Telangiectasia with Liver Cirrhosis Causing Portal Hypertension

**DOI:** 10.1155/2024/3574725

**Published:** 2024-01-17

**Authors:** Denisse Morales-Tovar, Froylan D. Martínez-Sánchez, Alejandro Gabutti-Thomas, Rodolfo Rivera-Martínez, Jacqueline Córdova-Gallardo

**Affiliations:** ^1^Facultad de Medicina, Universidad Nacional Autónoma de México, Escolar 411A, Copilco Universidad, Coyoacán, Ciudad de México 04360, Mexico; ^2^Department of Internal Medicine, Hospital General Dr. Manuel Gea Gonzalez, Calz. de Tlalpan 4800, Belisario Domínguez Secc 16, Tlalpan, Ciudad de Mexico 14080, Mexico; ^3^Department of Interventional Radiology, Instituto Nacional de Ciencias Medicas y Nutrición Salvador Zubiran, Vasco de Quiroga 15, Belisario Domínguez Secc 16, Tlalpan, Ciudad de México 14080, Mexico; ^4^Department of Imageology, Hospital General Dr. Manuel Gea Gonzalez, Calz. de Tlalpan 4800, Belisario Domínguez Secc 16, Tlalpan, Ciudad de Mexico 14080, Mexico; ^5^Department of Hepatology, Hospital General Dr. Manuel Gea Gonzalez, Calz. de Tlalpan 4800, Belisario Domínguez Secc 16, Tlalpan, Ciudad de Mexico 14080, Mexico

## Abstract

Hereditary hemorrhagic telangiectasia (HHT), also known as Rendu–Osler–Weber syndrome, is a vascular disorder of autosomal dominant etiology. The hallmark clinical feature is the presence of recurrent episodes of epistaxis in patients with vascular malformations and a tendency to bleed. We present the case of a 71-year-old woman who presented to the emergency department with upper gastrointestinal bleeding caused by esophageal varices, in conjunction with gastric angiodysplasias. The presence of oronasopharyngeal telangiectasias and hepatomegaly raised suspicion of HHT. The diagnostic workup confirmed the presence of angiodysplasia in the gastric region, portal arteriovenous malformation, and a pulmonary shunt.

## 1. Introduction

Hereditary hemorrhagic telangiectasia (HHT), also known as Rendu–Osler–Weber syndrome, is an autosomal dominant disorder with a prevalence of 1 case per 8,000–10,000 individuals [1]. It involves vascular malformations with direct communication between arterioles and venules, without the involvement of capillaries. This can result in shunting in solid organs and hollow viscera, as well as telangiectasias that can appear on the skin, nose, liver, brain, lungs, and digestive tract. It is estimated that up to 75% of patients may have hepatic involvement, although only 8–10% present with clinical manifestations related to this organ as the primary condition [1, 2].

## 2. Case Presentation

A 71-year-old woman with no relevant family history of genetic diseases was reported. She had a family history of a relative with hemorrhagic cerebrovascular disease and recurrent epistaxis as well as personal medical history of systemic arterial hypertension and diverticular disease confirmed by colonoscopy. She had no history of alcoholism, smoking, substance abuse, or other hepatotoxins. She presented with upper gastrointestinal bleeding, characterized by hematemesis and melena. On physical examination, she had telangiectasia in the nasogenian fold and hepatomegaly, with the liver edge palpable 1 cm below the costal margin.

### 2.1. Complementary Tests

The viral profile for hepatitis C antibodies and hepatitis B surface antigen was negative. Iron kinetics showed low levels of ferritin and transferrin saturation. Tests for antinuclear antibodies, anti-mitochondrial antibodies, and anti-smooth muscle antibodies were all negative. The remaining laboratory results are shown in Table 1. Furthermore, a diagnostic endoscopy was performed, revealing esophageal varices measuring 5 mm and gastric angiodysplasia in the gastric body, which was treated with argon plasma coagulation. Regarding the management of portal hypertension, a hepatic ultrasound was conducted, identifying signs of portal hypertension and splenomegaly, along with a possible portal arteriovenous malformation. Additionally, a transient elastography (FibroScan®) reported a liver stiffness measurement of 17.5 kPa (grade 4 fibrosis) and a controlled attenuation parameter reported a 369 dB/m return wave, indicating grade 3 steatosis.

**Figure 1:**
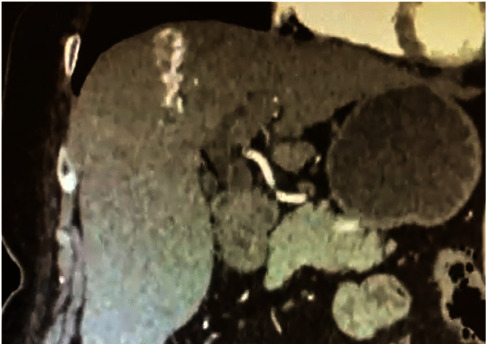
Quadriphasic abdominal tomography showing the arteriovenous fistula in segment VII during the arterial phase.

**Figure 2:**
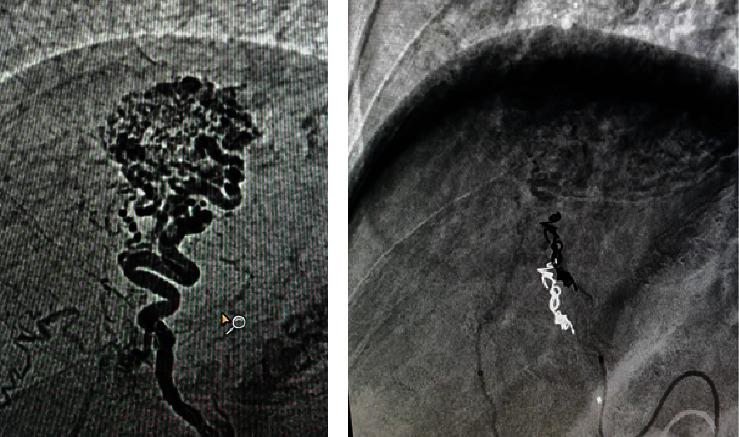
Angiography was conducted (a) before the embolization procedure to visualize the portal arteriovenous fistula and (b) after the procedure, during which 4 micro-coils measuring 2 × 3 mm × 2.3 cm and 2 measuring 2 mm × 4 cm were placed.

A portal arteriovenous malformation in segment VII was confirmed through quadriphasic abdominal tomography (CT) (Figure 1), and embolization was performed by interventional radiology (Figure 2). Since HHT was highly suspected, contrast-enhanced thoracic CT and brain magnetic resonance imaging (MRI) with gadolinium were conducted, showing no presence of pulmonary or cerebral arteriovenous malformations (AVMs). An echocardiogram revealed an intrapulmonary shunt due to the passage of bubbles into the left chambers following the administration of agitated saline solution into the right chambers.

### 2.2. Clinical Course

After the application of argon plasma, the gastrointestinal bleeding ceased. Furthermore, the patient started treatment for her microcytic hypochromic anemia due to chronic bleeding, resulting in a significant improvement in the patient's condition.

#### 2.2.1. Final Diagnosis

Hereditary hemorrhagic telangiectasia resulted in gastrointestinal bleeding of variceal origin, secondary to portal hypertension caused by both HHT and hepatic cirrhosis with an etiology of metabolic dysfunction-associated steatotic liver disease (MASLD). Additionally, there was a portal arteriovenous malformation in segment VII that was resolved through embolization, along with an intrapulmonary shunt.

## 3. Discussion

The case we are presenting is about a 71-year-old female patient who was hospitalized due to upper gastrointestinal bleeding, which manifested as hematemesis and melena. She had a family history of a relative with hemorrhagic cerebrovascular disease and recurrent epistaxis, as well as personal medical history of systemic arterial hypertension. There were no surgical history, alcoholism, smoking, or other substance abuse in her medical records. Upon physical examination, facial and nasal telangiectasias, as well as hepatomegaly, were identified. Additional tests revealed pancytopenia, including leukopenia, thrombocytopenia, and microcytic hypochromic anemia. Kidney function was normal, and liver function tests were mostly within the normal range, except for a slight elevation in gamma-glutamyl transpeptidase at 87 UI/L (normal range: 64 UI/L). Viral profiles were negative, iron kinetics and transferrin saturation were low, and tests for antinuclear antibodies, anti-smooth muscle antibodies, anti-LKM1, and anti-mitochondrial antibodies were all negative. An upper endoscopy was performed, which revealed 5 mm esophageal varices without signs of poor prognosis and an angiodysplasia in the gastric body. Electrocoagulation with argon plasma was applied to treat the angiodysplasia. The approach was further supplemented with vibration-controlled transient elastography (VCTE), which reported a stiffness measurement of 17.5 kPa (indicating fibrosis stage 4) and a controlled attenuation parameter (CAP) of 369 dB/m (indicating grade 3 steatosis). Subsequently, a contrast-enhanced abdominal triphasic tomography showed a portal arteriovenous malformation (AVM) in segment VII (Figure 1). An arteriography in interventional radiology revealed an arterioportal fistula connecting branches of the hepatic artery with branches of the portal vein. This fistula was embolized (Figure 2).

Hereditary hemorrhagic telangiectasia (HHT), also known as Rendu–Osler–Weber syndrome, is the second leading cause of hereditary bleeding disorders [1]. It is characterized by vascular malformations that can occur in the mucous membranes of the nose and the gastrointestinal tract, among other locations such as the brain, lungs, and liver [1, 2]. The most common hemorrhagic symptom is recurrent epistaxis (nosebleeds). In 97% of patients, the causal mutation is found in one of these genes: endoglin (ENG, HHT type 1) and activin receptor-like kinase 1 (ACVRL1, HHT type 2) [2].

The diagnosis is based on the Curacao criteria, which categorize it as “definite” with all three criteria met, “possible” with two criteria met, and “unlikely” with one or no criteria met [1]. Nonradiative imaging modalities are preferred for initial investigation. All patients should undergo pulmonary screening, including contrast transthoracic echocardiography followed by contrasted tomography, as well as imaging of the brain and liver [1, 2]. In cases of suspicion, deep venous thrombosis of the lower limbs should also be investigated.

Liver involvement occurs in 74%–79% of patients, with symptoms typically not manifesting before the third decade of life [1–3]. Thus, diagnosis usually occurs in adulthood. Only 8% of patients with liver involvement develop clinical symptoms [1]. The literature describes three types of intrahepatic shunts: arteriovenous, arterioportal, and portovenous [4]. Among these, arteriovenous shunts are the most frequent, and they are associated with hepatomegaly. In later stages, they can lead to congestive heart failure and pulmonary hypertension [5].

In our case, the patient met three diagnostic criteria for HHT: recurrent epistaxis, cutaneous and mucosal telangiectasias, and vascular malformations in the lungs and liver, as well as the diagnosis of hepatic cirrhosis demonstrated by VCTE, the etiology of which was nonalcoholic fatty liver disease (NAFLD). Thus, we concluded that the patient had both hepatic cirrhosis and HHT, which is a rare combination. There have been few published case reports of this association, which emphasizes the importance of conducting a detailed physical examination and complementary studies to rule out this disease in patients with clinical or familial data suggestive of HHT.

In cases of arterioportal fistulas, the primary clinical manifestation is the late development of portal hypertension, which becomes evident in the sixth or seventh decade of life, accompanied by ascites and variceal upper gastrointestinal bleeding [6]. In HHT, the liver can exhibit partial or diffuse regenerative activity, leading to nodular regenerative hyperplasia [6, 7]. The combination of fibrosis, nodular regenerative hyperplasia, and portal hypertension makes the diagnosis of cirrhosis challenging [7]. This condition may be a result of increased fibrous tissue in the portal spaces, possibly due to the proliferation of connective tissue layers of small vessels or the presence of microthrombi in this area, resulting in a histological pattern of atypical cirrhosis [8]. High-output heart failure is the most common cardiac symptom and is associated with vascular malformations that can be large enough to produce a palpable thrill or bruit in the epigastrium [7]. Elevated alkaline phosphatase and gamma-glutamyl transferase levels can lead to a misdiagnosis of cholecystitis.

The initial treatment is symptomatic. In cases of refractory epistaxis, tranexamic acid may be considered. Multiple transfusions may be required to maintain normal hemoglobin levels [8]. Iron transfusions are considered another option for anemia treatment. For telangiectasia treatment, laser therapy, sclerotherapy, radiofrequency ablation, and, as a last resort, bevacizumab or interferon can be considered [7, 8]. Esophageal varices should be managed according to standard protocols for upper gastrointestinal bleeding. Hepatic arterial embolization is an option for patients with arteriovenous and arterioportal shunts, where minimally invasive procedures are preferred [1, 7, 8].

Patients with symptomatic complications from hepatic venous malformations, particularly high-output refractory heart failure, biliary ischemia, or portal hypertension, may be candidates for transplantation. Performing a liver biopsy concurrently with arterial embolization in patients with hepatic venous malformations is generally not recommended due to the risk of bleeding [7, 8].

This association is rare, with only two reported cases in the literature [9, 10]. Therefore, sharing information about this clinical case contributes to current medical knowledge. The true prevalence is likely underestimated because it is an underdiagnosed condition, often due to the low suspicion arising from the nonspecific nature of the mostly asymptomatic clinical presentation. Genetic testing for pathogenic variants that alter vascular remodeling and are related to the presence of pulmonary hypertension, as in our patient's case, is uncommon. Therefore, diagnosis often relies on the Curacao criteria. In most cases, hepatic arteriovenous malformations may remain latent and, in some instances, can lead to heart failure, portal hypertension, pulmonary hypertension, and, very rarely, ischemic cholangitis. In asymptomatic individuals, screening for arteriovenous malformations and associated complications should be performed. For symptomatic patients, treatment of specific lesions should be undertaken, with monitoring and follow-up by internal medicine, gastroenterology, or hepatology for symptomatic cases.

Although DNA testing to identify mutations in the relevant genes (ENG, ACVRL1, and SMAD4) through methods such as DNA sequencing would have been very useful in the present case, it was not possible to perform for this patient. This limitation is noteworthy in the context of this case report.

## 4. Conclusions

In conclusion, this was a rare association of HHT with liver cirrhosis. HHT is the second most common cause of hereditary bleeding disorders. Meeting three Curacao criteria establishes the diagnosis. Initial screening involves searching for arteriovenous malformations in the brain, lungs, and liver. This article highlights the importance of a comprehensive approach and clinical diagnostic suspicion.

## Figures and Tables

**Table 1:** Biochemical data from the patient.

Laboratory tests	
Total leucocyte count (×10^3^/*μ*L)	5.5
Total lymphocyte count (×10^3^/*μ*L)	1.9
Total neutrophil count (×10^3^/*μ*L)	3.1
Hemoglobin (g/dL)	9.2
Hematocrit (%)	28.2
Mean corpuscular volume (Fl)	70.2
Mean hemoglobin concentration (pg)	22.4
Platelet count (×10^3^/*μ*L)	124
Fasting plasma glucose (mg/dL)	96
Blood urea nitrogen (mg/dL)	10.5
Serum creatinine (mg/dL)	0.7
Serum potassium (mEq/l)	4.2
Total cholesterol (mg/dL)	201
High-density lipoprotein cholesterol (mg/dL)	38
Low-density lipoprotein cholesterol (mg/dL)	150
Alkaline phosphatase (UI/L)	91
Gamma-glutamyl transferase (UI/L)	87
Aspartate aminotransferase (UI/L)	46
Alanine aminotransferase (UI/L)	26
Total bilirubin (mg/dL)	0.95
Prothrombin time (sec)	13.3
International normalized ratio	1.18
Partial thromboplastin time (sec)	33

## Data Availability

All data and materials are available from the corresponding author upon reasonable request.
